# Tracking interlinked microbial and geochemical succession over decades in landfilled municipal solid waste

**DOI:** 10.1128/aem.00311-26

**Published:** 2026-07-16

**Authors:** Kimber E. Munford, Daniel S. Grégoire, Laura A. Hug

**Affiliations:** 1 Department of Biology, University of Waterloo 8430, Waterloo, Ontario, Canada; 2 Department of Chemistry, Carleton University 6339, Ottawa, Ontario, Canada; University of Minnesota Twin Cities, St. Paul, Minnesota, USA

**Keywords:** landfill, municipal solid waste, landfill leachate, metagenomics, metals, biogeochemistry, leachate recirculation, microbial ecology

## Abstract

Landfills are heterogeneous built environments embedded in natural freshwater systems. They pose increasing risks of groundwater contamination from metal-bearing leachates over time. The interlinked succession of waste decomposition processes, microbial community membership, and metal cycling across a landfill’s lifespan has not been explored, reducing our ability to predict the long-term environmental impacts of landfills. Working with 1,647 metagenome-assembled genomes from a single landfill, from samples spanning over 39 years of waste decomposition, we identified changes in landfill biogeochemistry and connected these changes to shifts in microbial community composition and predicted functions over time. Comparing Older (aged 31–39 years) and Newer (aged 3–20 years) waste cells identified significant shifts in the availability of labile carbon, redox-associated processes, and concentrations of mobile metals—all higher in Newer cells. Newer cells were dominated by chemoorganoheterotrophs, while Older cells contained higher proportions of chemolithoautotrophs and organisms with higher metabolic versatility. Metal resistance and metal cycling genes were significantly more abundant in Older cells. Using geochemical data from the time of filling to the present and microbial membership data across six landfill cells of different ages, we developed a conceptual model of landfill characteristics across time. This model connects redox conditions and metal fate, highlighting leachate recirculation as a key process impacting many geochemical parameters and defining site chemistry. Our work highlights the substantial changes occurring over the stabilization phase and provides a conceptual model for understanding this critical, final stage in a landfill’s life cycle.

**IMPORTANCE:**

Aging landfills pose significant risks to environmental stability and are currently poorly modeled beyond ~20 years. Our examination of a single landfill across 39 years of waste degradation was a unique opportunity to examine the impact of time within a connected system. Our work connects geochemical data, microbial membership, and predicted function, as well as physical processes (e.g., leachate recirculation). Our conceptual model interlinks these facets across the lifespan of a landfill, providing an empirical data-based model of landfill aging. Previous models were extrapolated from younger waste and did not include the microbial dimension—a critical facet of the landfill ecosystem. Our model clarifies processes taking place in older wastes (30+ years), including oxygen infiltration, that have important implications for methane emission and metal mobility and fate over the longer term.

## INTRODUCTION

Landfills are utilized across the globe to store both residential and non-residential waste, including metals from construction waste, household refuse, and electronic waste. Landfills contribute to environmental and human health concerns over the course of their expected lifespans, including long-term methane emissions, punctuated and long-term groundwater contamination, and variable potential for leaching of heavy metals or other pollutants, under fluctuating redox conditions (1, 2).

Landfill refuse is generally buried in a sequential series of cells, which are each filled over a span of several years (2, 3) and expected to stably house the refuse for decades. Once closed, landfill cells go through a predictable set of biogeochemical phases as organic matter (OM) is decomposed by microbes. In phase 1 (aerobic), OM is rapidly metabolized through aerobic processes (3, 4). This phase is brief (1–2 years) as remaining oxygen is depleted via microbial respiration, leading to anoxic conditions. In phase 2 (anaerobic acid), hydrolytic, fermentative, and acetogenic bacteria continue metabolizing OM and produce organic acids, leading to decreases in pH and an overall decrease in OM (5). Phase 2 is followed by a rapid methanogenic phase (phase 3), where methane production peaks, then a slow methanogenic phase (phase 4). Methane production slows in phase 4 as labile OM sources are depleted and methanogenesis shifts to reliance on the downstream products from other organisms’ hydrolysis of polymeric carbon sources (1, 3, 6). The duration of phases 2–4 is highly variable and dependent on substrate conditions and climate, which impact the speed of OM breakdown (4, 6). Phase 4 can last for decades. It has been proposed that, beyond phase 4, landfills will enter a stabilization phase (phase 5). Some have defined this phase by the transition to microbial activity on more recalcitrant carbon sources (e.g., humic and fulvic acids) (4, 6–8). At this point, due to reduced microbial demand, oxygen entering landfill cells via passive diffusion can lead to increased oxygen availability and more oxidizing conditions.

Shifts in redox conditions over the landfill life cycle are expected to impact microbe-metal interactions and metal mobility as landfills age (3, 4, 9). Despite this, few have investigated the differences in biogeochemical conditions between older and newer landfill cells, particularly with respect to metal resistance and metal cycling (10, 11). Overwhelmingly, existing studies compare different landfills rather than cells within one landfill, which may impact comparisons of underlying chemistry (12). Additionally, they typically focus on carbon cycling and greenhouse gas production, omitting metals. Studies have found that the early stages of the landfill life cycle are characterized by pronounced (over 5–10 years) decreases in labile organic substrates (5, 6, 13). Others have shown that landfills experience a shift from microbial communities associated with the breakdown of OM to those dominated by lithoautotrophs over time (10, 14). What is less understood is how this microbial community succession impacts metal cycling and leaching. Some studies report higher metal concentrations in newer landfills, while others predict that metals may be mobilized in the later phases of the landfill life cycle (3, 10, 11). Increasingly oxidizing conditions in phase 5 can lead to oxidation of reduced sulfides, releasing metals and reducing pH (3, 15). Additionally, organic substrates (e.g*.,* humic or fulvic substances) remaining in waste during the stabilization phase (phase 5) can form soluble complexes or colloids with metals, increasing metal mobility (3, 4).

Here, we investigate microbial community structure and associated functional genes between Older (aged 31–39 years) and Newer (aged 3–20 years) landfill cells from a landfill in the Northeastern U.S. We connect these examinations of metabolic guilds and microbe-metal interactions with changes in leachate chemistry collected over more than three decades. A previous study by Grégoire et al. (1) looked at microbiological and geochemical evidence for methane cycling in the same system, where they highlighted oxygen infiltration into older landfill cells. They also noted the potential impact of leachate recirculation on geochemistry and redox conditions, since leachate recirculation occurred sporadically from 1985 to 2009.

Our study assessed differences in microbial communities and leachate geochemistry over time, focusing on major microbial metabolic processes involved in and controlled by redox cycling. We hypothesized that older landfill cells would host microbial communities whose functional genes had a higher potential for utilization of inorganic electron acceptors and cycling of metals, while newer landfill cells would have microbial communities with functional genes more strongly associated with anaerobic fermentative processes.

## RESULTS

### Site description and sampling

The sampled site is a municipal landfill located in the northeastern United States (NEUS), site anonymity requested by site engineers. The site is composed of six landfill cells (A, B, C, D, E, and F), two of which are divided into sub-cells (D into D1 and D2, and F into F1 and F2), each equipped with leachate collection systems. Landfill cells were broadly separated by age into “Older” (cells A, B, and C; aged 31–39 years), “Intermediate” (cells D1 and D2; aged 24–26 years), and “Newer” (cells E, F1, and F2, 3–20 years) based on their predicted landfill phase (Older = phases 4 and 5, Newer = phases 2 and 3). Landfill phases were determined by Grégoire et al. (1) in their work investigating methane cycling at the site, utilizing measured biological oxygen demand (BOD) and chemical oxygen demand (COD) to assign phases. Leachate samples were collected in 2019 for sequencing and metagenomic analysis (see Materials and Methods [1]). The site owners enthusiastically provided historic and current leachate geochemistry records under a Freedom Of Information Act (FOIA) request. Notably, microbial samples come from a single point in time (sampling in 2019), whereas leachate chemistry data spans the lifetime of the cells. As a result, microbial analyses focus on comparisons between the oldest (“Older”) and youngest (“Newer”) cells. Integrations of microbial and leachate chemistry data draw on the entire data set.

### Taxonomic composition of microbial communities

**Fig 1:**
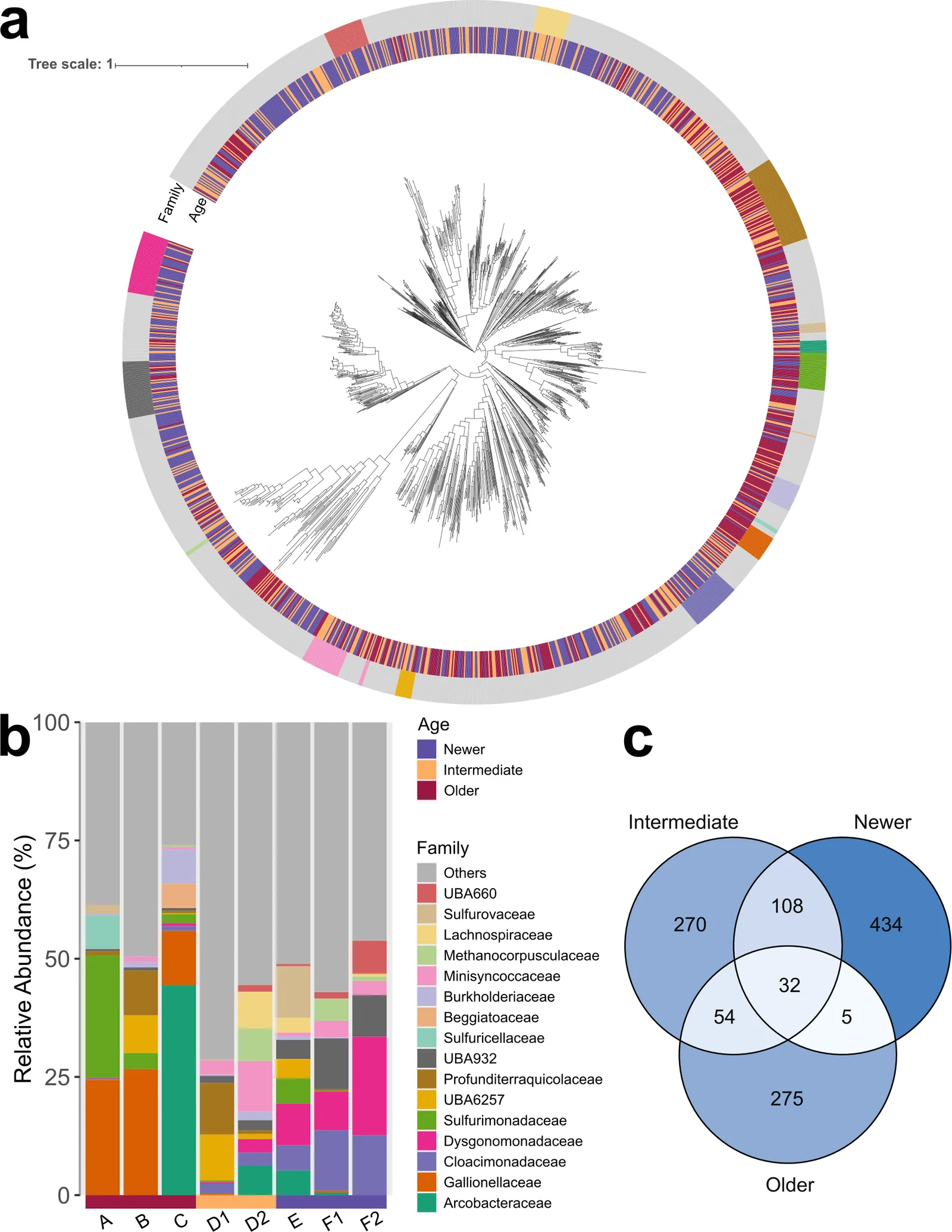
(**a**) Phylogenomic tree based on 25 target gene hidden Markov models (HMMs) for bacteria and archaea, placing 1,570 out of 1,647 MAGs in relation to each other, generated in GToTree v1.6 (16). Rings depict outer to inner (1) family-level taxonomy, (2) cell age category. MAGs in the tree were not dereplicated so that sample-specific relative abundances could be depicted. Colors for families correspond to those in the (**b**) barplot of relative abundance of all bacterial and archaeal families with over 5% relative abundance in at least one landfill cell, with lower abundance lineages grouped under “Other.” Taxonomy was taken from GTDB-tk r266, where lineages starting with UBA are officially recognized but not yet formally named. (**c**) Venn diagram indicating counts and percentages of shared MAGs (clustered at 99% similarity using DRep) among age categories across landfill cells. Venn sections are colored by proportion of MAGs within a category, with higher values corresponding to darker colors.

Starting from 1,647 total bins, dereplication was performed at 99% similarity to compare shared populations across cells, yielding 1,178 metagenome-assembled genomes (MAGs) across all eight metagenomes (all cells and sub-cells). Fewer total MAGs (>70% completion, <10% contamination) were detected in Older landfill cells (138 ± 48) than Intermediate (245 ± 34.7) or Newer cells (248 ± 42.7), with 93 MAGs in cell A, 188 in cell B, 134 in cell C, 220 in D1, 269 in D2, 210 in E, 294 in F1, and 239 in F2. Only 32 dereplicated MAGs were shared among all three age groups, and only five dereplicated MAGs were shared between Older and Newer cells to the exclusion of Intermediate cells (Fig. 1c).

Beyond limited shared MAGs, there were large taxonomic differences between communities from Older and Newer cells, based on taxonomic assignment by GTDB-tk (release 226) (17) and from a phylogenomic tree encompassing MAGs from all samples (Fig. 1a). Taxonomic assignment, trees, and all subsequent analyses were performed using the set of 1,647 non-dereplicated MAGs to capture cell-by-cell differences (see Materials and Methods). Of the 1,647 MAGs in the data set, only 77 had too few hits to the required marker genes to be included in the tree. MAGs that were removed had a relative abundance of <0.5% on average. Most of these 77 MAGs (63.6%) were within the Halobacteriota (22 MAGs) or placed within groups with small genomes that may be missing key markers (i.e*.,* Patescibacteriota [18 MAGs], Nanobdellota [seven MAGs], and Micrarchaeota [two MAGs]). A table of taxonomic classifications, relative abundance, tree membership, completeness and contamination, and dereplication cluster membership is available on the Open Science Framework (OSF) (https://doi.org/10.17605/OSF.IO/MSDB8) (18).

Overall, Older cells had lower Shannon Diversity (3.70 ± 0.640) than Intermediate (4.94 ± 0.0322) or Newer (4.63 ± 0.0445) cells. Older cells had a strong enrichment in members of the families Gallionellaceae and Sulfurimonadaceae, with enrichment in Arcobacteraceae in cell C. Collectively, members of the Gallionellaceae, Sulfurimonadaceae, and Arcobacteraceae represented 50.3% relative abundance in cell A, 30.0% in cell B, and 57.8% in cell C (Fig. 1b), highlighting the overall lower microbial diversity in Older cells. The Gallionellaceae family consisted of 15 dereplicated MAGs across one unidentified (JAQTOM01 [one MAG]) and two identified genera (*Gallionella* [10 MAGs] and *Sideroxyarcus* [four MAGs]), and comprised 24.4% (three MAGs), 26.7% (seven MAGs), and 11.5% (six MAGs) in cells A, B, and C, respectively. Members of the Gallionellaceae were also present in D1 (0.327% [two MAGs]) and F1 (0.371% [one MAG]), but at lower abundance than in Older cells. The family Sulfurimonadaceae was represented by 24 dereplicated MAGs across two genera (*Sulfuricurvum* [12 MAGs] and *Sulfurimonas* [12 MAGs]) in all cells (Fig. 1b; Table S1), and comprised 25.9% (six MAGs), 3.36% (six MAGs), and 1.91% (seven MAGs) in cells A, B, and C, respectively. Relative abundance of members of the Sulfurimonadaceae was <1% in all Intermediate and Newer cells (one MAG in D1, one in D2, eight in E, and one in F1). Arcobacteraceae was the most abundant family in any one sample, comprising 44.4% of the community in cell C. Within cell C, three dereplicated Arcobacteraceae MAGs were present, two from the genus *Aliarcobacter* (44.0%) and one unidentified (CAIJNA01 [0.495%]). Arcobacteraceae were not detected in cells A or B but were present at lower abundances in cells D2 (6.22% [four MAGs]), E (5.19% [one MAG]), and F1 (0.548% [one MAG]).

Contrastingly, Newer cells showed enrichments in members of the families Dysgonomonadaceae and Cloacimonadaceae. Dysgonomonadaceae consisted of 17 dereplicated MAGs across two identified genera (*Proteiniphilum* [10 MAGs] and *Petrimonas* [two MAGs]) and five MAGs unidentified at the genus level. Relative abundance of MAGs in the genus *Proteiniphilum* was 3.04% (five MAGs), 7.45% (seven MAGs), and 17.2% (10 MAGs) in E, F1, and F2, respectively. Relative abundance was below 1% in Older cells (three MAGs in C only) and D1 (one MAG), but slightly more abundant in D2 (2.32% [three MAGs]). Relative abundance of MAGs in the genus *Petrimonas* was 2.12% (five MAGs), 0.392% (seven MAGs), and 2.00% (10 MAGs) in E, F1, and F2, respectively, and less than 1% in Older (one MAG in A and three in C) and Intermediate (one MAG in D1 and 3 in D2) cells. In total, the relative abundance of all unidentified members of the Dysgonomonadaceae family was 3.66% (three MAGs), 0.319% (two MAGs), and 1.61% (four MAGs) in E, F1, and F2, respectively, and they were only detected in one other cell (D2 at 0.393% [two MAGs]). The family Cloacimonadaceae consisted of 18 dereplicated MAGs, most of which were unidentified at the genus level (UBA5456 [10 MAGs]), with the identified genera being *Syntrophosphaera* (four MAGs) and *Cloacimonas* (four MAGs). Relative abundance of members of the Cloacimonadaceae was 5.38% (10 MAGs), 12.8% (nine MAGs), and 12.7% (six MAGs) in E, F1, and F2, respectively. Cloacimonadaceae abundances were lower in Intermediate cells, with 2.21% in D1 (three MAGs) and 2.80% in D2 (six MAGs), and at less than 1% in all Older cells (one MAG in A and two in C).

As shown above, Intermediate samples tended to be variable, with microbial composition in D1 more strongly resembling Older cells, while composition in D2 was closer to Newer cells. Interestingly, both subcells of cell D were enriched in families whose members are typically very small cells (≤0.8 µm) with reduced genomes (19, 20). This included the family Profunditerraquicolaceae (D1: 10.9% and D2: 0.749%) with 43 MAGs from two identified genera (*Undivivens* [D1: 0.449%, D2: 0.242%] and *Sherwoodlollariibacterium* [D1:0.353%, D2: 0.241%]), each represented by one dereplicated MAG, and 41 MAGs from unidentified genera (D1: 10.14% [24 MAGs] and D2: 0.266% [two MAGs]). Members of the Profunditerraquicolaceae were also found in Older (30 MAGs in A, B, and C) and Newer (two MAGs in F1 only) cells at less than 1% relative abundance in all cells except B, where relative abundance was 9.47% (22 MAGs).

Two families within the Patescibacteriota phylum were also enriched in the Intermediate (D) cells. Members of the Minisyncoccaceae, represented by 17 MAGs across two identified genera (*Microsyncoccus* [three MAGs] and *Minisyncoccus* [two MAGs]) and four unidentified genera (12 MAGs), had 2.94% (five MAGs) and 10.6% (eight MAGs) relative abundance in D1 and D2, respectively. Members of Minisyncoccaceae were also present in Newer cells, with relative abundances of 0.762% (one MAG), 3.44% (six MAGs), and 2.97% (five MAGs) in E, F1, and F2, respectively. Relative abundance of Minisyncoccaceae was less than 2% in all Older cells, detected only in B and C with three MAGs in each cell. Finally, an uncultured order (UBA6257) in the Patescibacteriota phylum (18 MAGs) had high relative abundance in cell D, with seven MAGs in D1 and three MAGs in D2, accounting for 9.61% and 1.04% relative abundance, respectively. MAGs from UBA6257 were also present in Older (B: 8.75% [four MAGs], C: 0.248% [one MAG]) and Newer (E: 5.45% [seven MAGs], F1: 0.723% [one MAG], and F2: 0.178% [two MAGs]) cells.

Although there were some similarities at the Family level, few dereplicated MAGs (five total) were shared between Older and Newer cells (Fig. 1c). In contrast, 42% of dereplicated MAGs in Intermediate cells were shared with Newer, Older, or both. Thus, Intermediate cells reflect a transitionary state between Older and Newer, highlighting how few taxa can persist following transition between landfill phases. Due to the clear distinction between microbial community compositions in Older and Newer cells, we opted to focus on comparisons between these two age groups for our downstream analyses.

### Microbial diversity and functional genes

There were substantial differences in the predicted functional capacity of microbial communities in landfill cells of different ages. In total, we examined 200 different functional gene groups and pathways. We utilized Distilled and Refined Annotation of Metabolism (DRAM) v1.0 (21) to annotate MAGs, focusing on 93 functional gene groups and pathways, covering central metabolism, carbohydrate-active enzymes (CAZymes), conversion of short-chain fatty acids (SCFAs) and alcohols, methanogenesis and methanotrophy, nitrogen cycling, and sulfur cycling. Annotations from four functional groups were identified using FeGenie (22), focusing on iron cycling. Ten more were annotated with an arsenic (As) metabolism and resistance pipeline from Dunivin et al. (23), and 93 were derived from BacMetScan (24), covering microbial metal interactions (respiration and resistance). Genes that contributed to functional gene categories across the different annotation tools are listed in Table S2. Only genes that were significantly different at *P* < 0.05 are reported here.

To investigate differences in functional potential between cells of differing ages, we integrated all annotations into a presence-absence table for all functional genes, groups, or pathways (available on the Open Science Framework at https://doi.org/10.17605/OSF.IO/MSDB8) (18). DRAM estimates coverage of metabolic pathways and completion of electron transport chain (ETC) complexes based on the percentage of genes recovered. For our presence-absence assessment, we considered pathways and ETC complexes to be present when DRAM’s estimate was >70% for a given MAG. Then, the abundance of functional genes was calculated by summing the relative coverage of all MAGs that possessed a given functional gene, group, or pathway within a cell, representing the proportion of the microbial community with the encoded potential for a given function.

Across the full data set of 1,647 MAGs, only six MAGs (from 0.107% to 0.730% relative abundance) had no functional gene hits with the 70% completion cutoff, including for central metabolic pathways. Four of these MAGs were members of the Patescibacteriota phylum, and two were members of the archaeal phylum Nanobdellota (formerly “Nanoarchaeota”). Members of both groups live as symbionts on prokaryotic hosts, lacking portions of some pathways essential for independent life (20, 25, 26). Genomes for these organisms ranged from 70.8% to 78.5% completion, although these groups often lack many of the universal single-copy marker genes used to estimate completeness. Therefore, it is possible that core metabolic genes were genuinely absent, but it is also possible that these genes were not captured.

We investigated differences in functional gene and pathway abundance and prevalence between Older and Newer cells using Wilcoxon rank-sum tests (*P* < 0.05) (Table S2). To explore the fermentative capacity of the microbial communities, we investigated the presence or absence of Complexes I–V of the electron transport chain and DRAM annotations associated with the breakdown of SCFAs and carbohydrates (CAZymes).

Complex IV of the ETC is associated with the transfer of electrons to oxygen, the final electron acceptor in aerobic respiration, so a lack of this complex indicates likely anaerobic respiration. The absence of Complexes I–IV of the ETC indicates that an organism is likely an obligate fermenter. Overall, we found that seven out of eight Complexes I–IV of the electron transport chain were significantly more abundant in Older cells, including both low- and high-affinity oxidases, indicating that variable oxygen availability in the system supports aerobic metabolisms (Table S2 and Fig. S1). The relative lack of Complexes I–IV in Newer cells points to fermentative metabolisms and anaerobic respiration as dominant in Newer cells. ATPases also point to differences in metabolic processes between cell ages. F-type ATPases, which were significantly more abundant in Older cells, often couple to an ETC for oxidative phosphorylation, pointing to aerobic respiration when Complexes I–IV are also present. In Newer cells, V/A-type ATPases were significantly more abundant, possibly reflecting maintenance of proton gradients in microbes with low respiratory activity.

**Fig 2:**
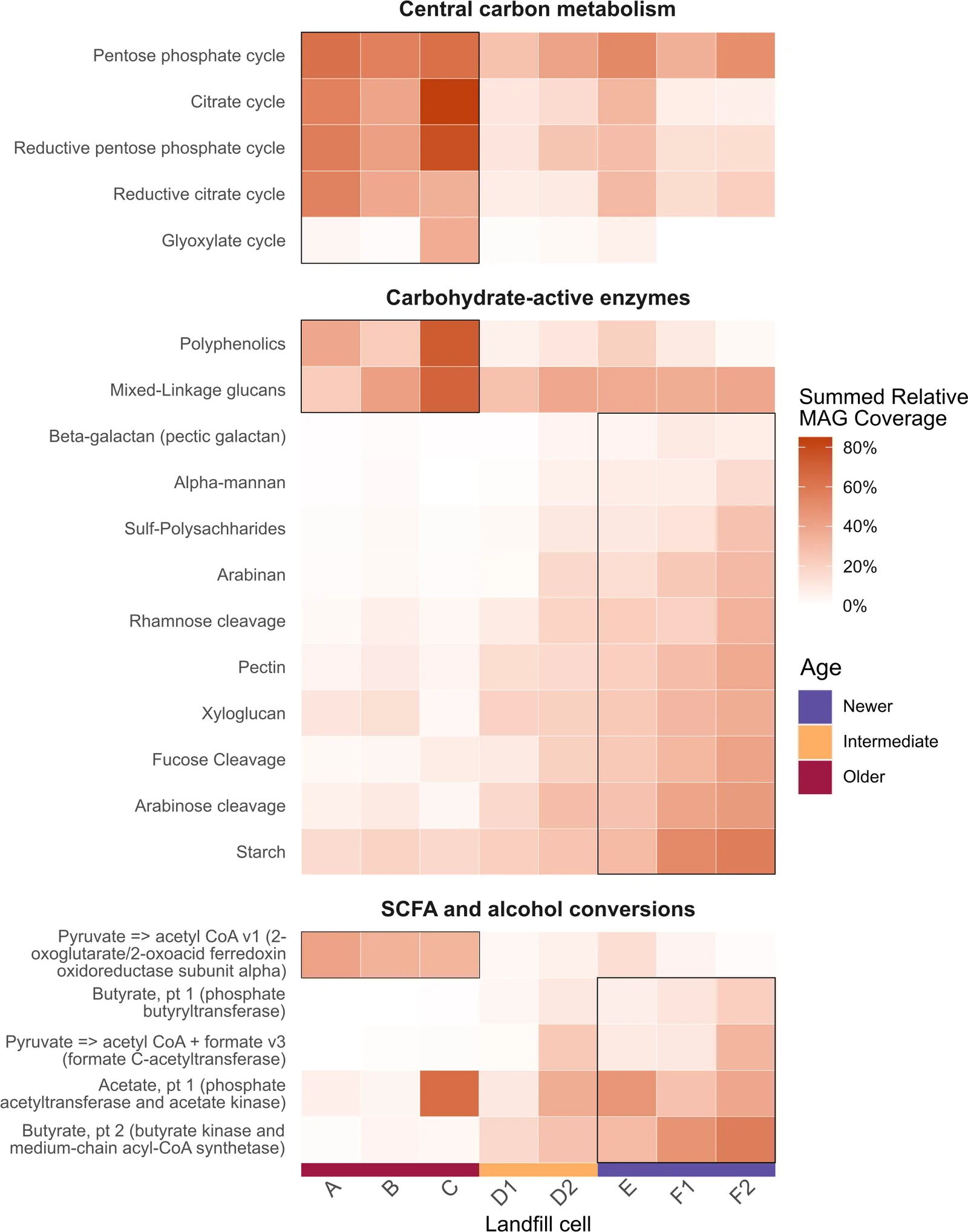
Carbon cycling differential abundance across landfill cells. Heatmap depicting the summed relative coverage of all MAGs in each landfill cell that contain genes associated with specific functions within carbon cycling. Only genes/pathways/processes which had significantly different abundances between Older and Newer cells (Wilcoxon rank-sum test, Benjamini-Hochberg-corrected *P*-value of < 0.05) are displayed. Boxes indicate whether abundance was significantly higher in Newer or Older cells. Central C metabolism pathways from DRAM were required to be >70% complete within a MAG for inclusion. Carbohydrate-active enzymes from DRAM either indicate the presence of genes for both backbone and oligomer cleavage (alpha-mannan, arabinan, beta-galactan, mixed-linkage glucans, pectin, starch, sulf-polysaccharides, and xyloglucan) or only oligomer cleavage (arabinose cleavage, fucose cleavage, and rhamnose cleavage). For polyphenolics, multiple oxidative genes are accounted for. For DRAM SCFA and alcohol conversions, the gene(s) of interest are listed in brackets. Further information can be found in the DRAM documentation.

Functional gene groups associated with central carbon metabolism and OM decomposition also indicated that fermentation was the key metabolic process in Newer cells compared to autotrophy in Older cells (Fig. 2). Older cells had significantly higher abundance of genes or gene groups associated with the reductive pentose phosphate (Calvin) cycle (Older: 59.5% ± 17.7%; Newer: 19.3% ± 8.47%), reductive citrate (Arnon-Buchanon) cycle (Older: 43.1% ± 10.9%; Newer: 22.5% ± 7.99%), glyoxylate cycle (Older: 14.2% ± 19.4%; Newer: 2.12% ± 3.46%), and citrate (Krebs) cycle (Older: 60.3% ± 22.4%; Newer: 15.4% ± 14.7%), suggesting the importance of both aerobic and anaerobic respiration, as well as carbon fixation in Older cells (Fig. 2). Notably, across all samples, genes for the reductive acetyl-CoA (Wood-Ljungdahl) pathway, associated with anaerobic carbon fixation, were low abundance, at 1.09% ± 1.36% in Older cells and 2.75 ± 1.02% in Newer cells.

Contrastingly, Newer cells had significantly higher abundance of gene groups associated with the breakdown of carbohydrates. These included CAZymes for breaking down complex carbohydrates like pectin, starch, mannans, and xyloglucan, with 10/16 identified CAZymes occurring at significantly higher proportions in Newer cells. The only exceptions were CAZymes for polyphenolics and mixed-linkage glucans, which were significantly more abundant in Older cells. The relatively higher abundance of CAZy genes for polyphenolics and mixed-linkage glucans in Older cells may point to more complex carbon sources persisting in Older cells. Additionally, Newer cells had a higher abundance of some genes and gene groups associated with the breakdown of SCFAs and alcohols (5 out of 11 genes were significantly different), such as acetate, butyrate, and pyruvate (Fig. 2). Enrichment in CAZy genes and genes associated with SCFA breakdown further point to fermentative activity in Newer cells.

Finally, there were also significant differences in genes and gene groups associated with methanogenesis and methanotrophy between Older and Newer cells (Table S2 and Fig. S2). Briefly, genes associated with methane oxidation were significantly more abundant in Older cells, further indicating possible oxygen infiltration alongside slow methanogenesis in Older cells, though overall relative coverage of MAGs with those genes was low (<2%) (Fig. S2). Grégoire et al. (1), who explored methanogenesis in this site, also observed a decline in putative methanogens with cell age.

**Fig 3:**
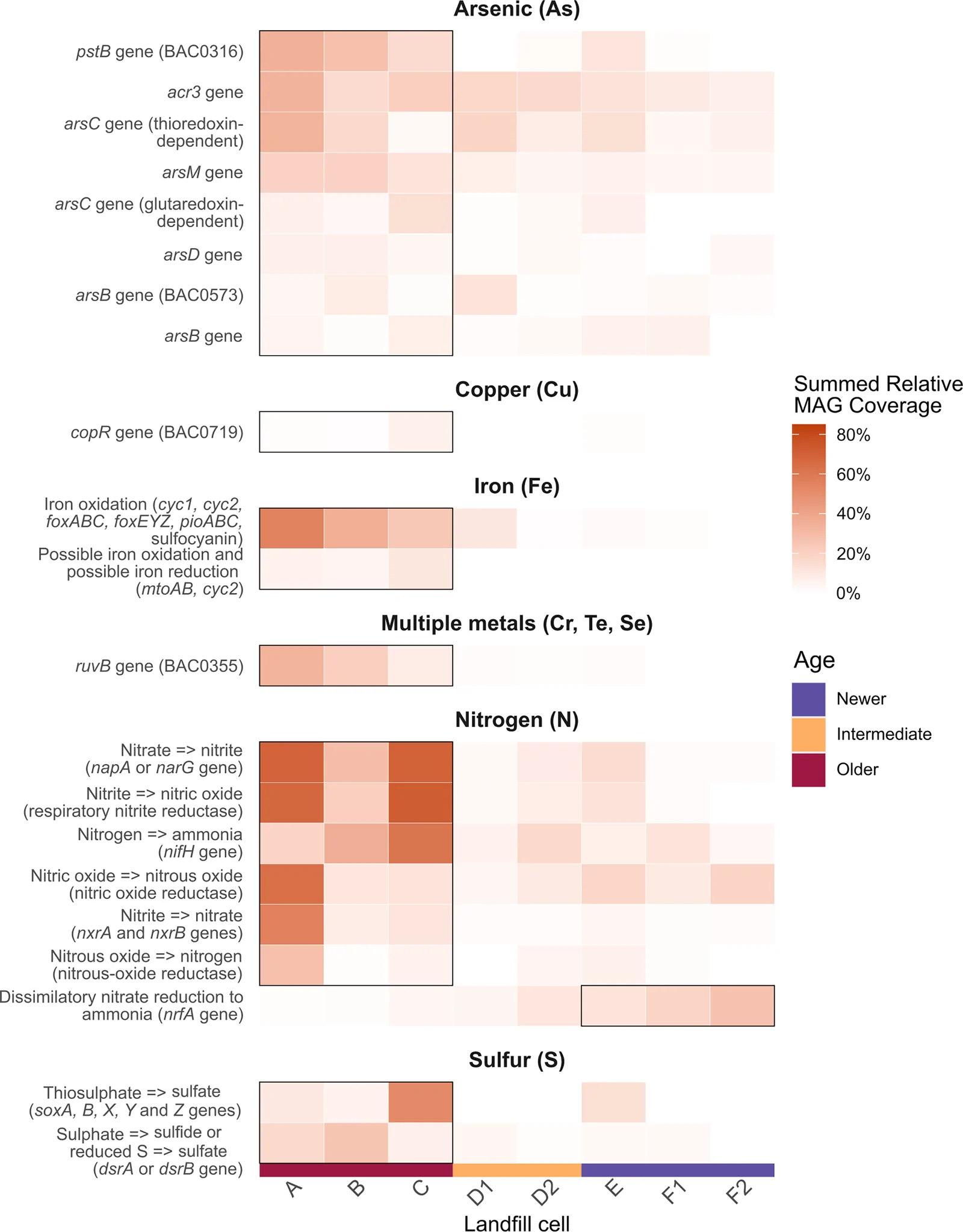
Redox cycling and metal resistance differential abundance across landfill cells. Heatmap depicting the summed relative coverage of all MAGs in each cell that contain genes/processes associated with specific functions within redox cycling and metal resistance. Only genes/processes that had significantly different abundances between Older and Newer cells (Wilcoxon rank-sum test, Benjamini-Hochberg-corrected *P*-value of < 0.05) are displayed. Genes/processes with a maximum of <0.05% relative coverage were pruned for clarity and are presented in Fig. S3. Genes and functional groups come from four tools: FeGenie and an HMM set from Dunivin et al. for Fe and As, respectively, BacMetScan for metal-resistance genes, and DRAM for functional groups associated with S and N cycling. For As-related genes detected using the Dunivin et al. HMM set, the gene of interest is listed. For BacMetScan annotations, the BacMet number is listed in brackets following the gene. For FeGenie annotations, the process is listed, followed by the relevant genes in brackets. Finally, for DRAM annotations, the process is listed, followed by the gene(s) of interest in brackets.

Older cells had a higher abundance of MAGs predicted to utilize a variety of alternative electron donors and acceptors. Based on DRAM annotations (for nitrogen [N] and sulfur [S]) and FeGenie results (for iron [Fe]), we found that functional genes and gene groups for reduction and oxidation of all three elements were significantly more abundant in Older cells, pointing to metabolic flexibility in Older cells (Fig. 3). Notably, genes associated with all steps of denitrification (nitrate → nitrite [*napA* or *narG* gene] → nitric oxide [respiratory nitrite reductase gene] → nitrous oxide [nitric oxide reductase gene] → nitrogen [nitrous-oxide reductase gene]) were significantly more abundant in Older cells, with abundance of these genes ranging from 11.5% to 55.9% in Older cells and from 2.63% to 15.5% in Newer cells (Fig. 3). Similarly, the abundance of the functional gene groups associated with the oxidation of nitrite to nitrate (*nxrA* and *nxrB* genes) (Older: 25.2% ± 26.9%; Newer: 15.5% ± 5.1%) and bacterial/archaeal ammonia oxidation (*amoA* gene) (Older: 1.96% ± 1.71%; Newer: 0.340% ± 0.588%) were also significantly higher in Older cells. One exception was dissimilatory nitrate reduction to ammonia (*nrfA* gene), which was significantly more abundant in Newer cells (Older: 1.96% ± 1.76%; Newer: 19.3% ± 7.47%). Finally, the abundance of the nitrogen fixation gene *nifH* was significantly higher in Older cells (Older: 38.7% ± 21.4%; Newer: 7.61% ± 4.13%). Taken together, the primary processes for generating biologically available nitrogen differ between cell age categories. There was evidence for both aerobic and anaerobic processes (including N fixation) in Older cells, whereas genes for dissimilatory nitrate reduction to ammonia were more abundant in Newer cells. For example, 60% of MAGs with genes encoding respiratory nitrite reductase also had high-affinity oxidases (compared to 8% of all MAGs), pointing to respiratory flexibility in the fluctuating redox conditions of Older cells. These results further highlight that changes in nitrogen metabolism track shifts in redox conditions over the landfill life cycle.

Functional gene groups for the reduction and oxidation of S and Fe followed similar trends. Two functional gene groups for the oxidation or reduction of sulfate were significantly different in abundance between Older and Newer cells. The functional gene group for partial or complete oxidation of thiosulfate to sulfate (including *soxA, soxB, soxX, soxY,* and *soxZ*) was highly abundant in Older (22.7% ± 25.5%) cells, in contrast to Newer (4.70% ± 8.14%) cells. Interestingly, the functional gene group for dissimilatory reduction or oxidation of sulfate (*dsrA* or *dsrB*) was also much higher in Older (16.5% ± 9.33%) than Newer (1.84% ± 1.35%) cells (Fig. 3). Functional gene groups identified by FeGenie as associated with iron oxidation (Older: 38.5% ± 15.5%; Newer: 0.875% ± 1.00%) and possible iron oxidation or reduction (Older: 7.09% ± 2.92%; Newer: 0.890% ± 0.154%) were also significantly more abundant in Older cells (Fig. 3 and Table S2). The increase in sulfate and iron reduction genes in Older cells relative to nitrate reduction genes in Newer cells may reflect shifts in terminal electron acceptors as substrates are depleted over time. Finally, the abundance of arsenic metabolism genes (e.g., *arrA*, *aioA*) was not significantly different between Older and Newer cells across either Dunivin et al.’s HMM set (23) or via the BacMet database. The heightened abundance of genes or gene groups for both oxidation and reduction of Fe, S, and N compounds in Older cells highlights a greater reliance on alternative electron donors and acceptors in Older cells.

Although there were no significant differences in the abundance of respiratory arsenic metabolism genes, genes associated with arsenic detoxification and tolerance were significantly different. The abundance of the arsenic methyltransferase *arsM* (Older: 17.3% ± 4.30%; Newer: 4.30% ± 1.23%) and two cytoplasmic arsenate reductase genes (thioredoxin-dependent and glutaredoxin-dependent) were also significantly higher in abundance in Older cells. Thioredoxin-dependent arsenate reductase was more abundant in general (Older: 17.8% ± 15.3%; Newer: 7.80% ± 5.46%) than glutaredoxin-dependent arsenate reductase (Older: 8.06% ± 5.27%; Newer: 2.41% ± 3.88%). Both are associated with the cytoplasmic reduction of As(V) to As(III) so it can be pumped out of the cell via an arsenite efflux pump (*acr3* or *arsB*). Both arsenite efflux pump genes were significantly more abundant in Older cells, with arsenical resistance-3 protein (*acr3*) (Older: 23.6% ± 8.96%; Newer: 9.78% ± 2.95%) being more abundant than *arsB*, which was detected through both Dunivin et al.’s HMM set and BacMetScan. The two annotation tools use different methods to annotate genes (23, 24), and there was little overlap in the annotation of *arsB* between the two methods, with 70 MAGs identified as containing *arsB* from Dunivin et al.’s set and 99 from BacMet, but only four overlapping between the two. Across both annotation tools, the abundance of the gene encoding the arsenite efflux pump (*arsB*) ranged from 1.31% to 8.43% in Older cells and from 0.266% to 5.99% in Newer cells. The arsenic metallochaperone (*arsD*) is also significantly more abundant in Older cells (Older: 5.76% ± 1.39%; Newer: 1.84% ± 1.55%) and chaperones intracellular As(III) to *arsB*-encoded efflux pumps. A higher relative abundance of genes that encode various parts of the arsenic detoxification system suggests more prevalent arsenic resistance in Older cells than in Newer cells. Additionally, the phosphate-transporting ATPase (*pstB*), which can bring As(V) into cells, was also significantly more abundant in Older cells (Older: 26.1% ± 9.30%; Newer: 4.36% ± 6.21%), indicating the possibility for increased uptake of arsenic in Older cells as a consequence of phosphorus scavenging.

We also looked for other metal-resistance genes using BacMetScan and the BacMet database, finding that Older cells displayed significantly higher abundance of genes associated with mitigating chromate toxicity (*chrA*, *chrF*), copper resistance and metabolism (*copR*), cobalt-zinc-cadmium resistance (*czcA*), cobalt, cadmium, and nickel transport (*dmeF*), tellurium resistance (*terD*), and repairing DNA damage following chromate, tellurite, or selenite exposure (*ruvB*) (Fig. 3; Fig. S3). The DNA repair gene encoding ATP-dependent DNA helicase (*ruvB*) was the most abundant of these metal resistance genes (Older: 21.1% ± 12.8%; Newer: 0.660% ± 0.843%). All the other metal resistance genes listed above were below 3% relative coverage, and, interestingly, five of six of these (*chrA*, *chrF*, *czcA*, *dmeF*, and *terD*) were not found in any MAGs from Newer cells. The gene encoding the copper resistance regulator (*copR*) was also below 3% abundance on average but present in both Older (2.66% ± 3.10%) and Newer (0.404% ± 0.495%) cells. Enrichment of metal-resistance and DNA repair genes in Older cells may point to mitigation of toxicity being an important survival strategy as landfill cells age.

### Leachate chemistry

Leachate chemical parameters were investigated for the 5 years prior to sampling and the period of filling for individual cells. Key parameters were selected that associate with microbial functions of interest (Table 1, from full set in Table S3). Tables of all leachate chemistry parameters over time have been uploaded to the Open Science Framework (https://doi.org/10.17605/OSF.IO/MSDB8) (18). Further supporting the differential abundance of redox-cycling genes discussed above, conditions were less reducing in Older cells relative to Newer cells (Table 1). BOD and COD were both significantly lower in Older cells in the five years prior to sampling, and there was a massive decline in both BOD (from 9,629 ± 9,940 to 71.6 ± 194.2 mg/L) and COD (from 14,285 ± 13,298 to 446 ± 387 mg/L) since the time of filling, indicating a clear decrease in labile carbon sources (Table 1 and Table S3). Total organic carbon (TOC) in leachate was also significantly lower in Older cells in this time window. Other carbon sources were significantly lower in Older cells, including total phenolics, acetic acid, propionic acid, and both SCFAs. The other investigated SCFAs were not statistically significantly different but had overall lower concentrations in Older cells (Table S3). Shifts in BOD, COD, and labile carbon sources over time and between Older and Newer cells align with differences in abundance of functional genes related to fermentation and carbon metabolism over time. Interestingly, leachate concentrations of metals (As, Cr, Fe, and Ni) and redox-associated parameters (reduced sulfide and ammonia) were significantly higher in Newer cells than Older cells in the five years prior to sampling, despite lower abundance of functional genes associated with sulfate reduction and metal resistance in Newer cells (Table S3). The lower metal concentrations in leachate from Older cells may be linked to the formation of iron oxyhydroxide phases under less reducing conditions, consistent with the enrichment in Fe-oxidation genes in Older cells.

**TABLE 1:** All geochemical measurements (mean ± sd) for key variables, collated by landfill cell age class during filling of the cells and during the 5 years prior to sampling in winter 2019^*a*^

Analyte	Older (A, B, and C)	Newer (E, F1, and F2)	Benjamini-Hochberg corrected *P* value
Conductivity (μs/cm)	4,665 ± 2,083	7,789 ± 3,629	<0.001
Redox (mV)	−36.1 ± 71.8	−88.8 ± 69.5	<0.001
Bicarbonate (mg/L)	1,557 ± 774	1,842 ± 1,242	0.00257
BOD_5_ (mg/L)	71.6 ± 194.2	251 ± 308	<0.001
COD (mg/L)	446 ± 387	1,052 ± 875	<0.001
TOC (mg/L)	115 ± 56.2	272 ± 227	<0.001
Sulfide (mg/L)	0.845 ± 0.471	5.17 ± 11.4	<0.001
Ammonia-N (mg/L)	219 ± 123	458 ± 306	<0.001
Acetic acid (mg/L)	12.2 ± 25.1	61.3 ± 71.4	<0.001
Propionic acid (mg/L)	9.99 ± 21.5	34.2 ± 72.4	0.0151
Total phenolics (mg/L)	0.173 ± 0.183	0.835 ± 1.59	<0.001
Arsenic (μg/L)	16.9 ± 14.5	61.4 ± 61.4	<0.001
Chromium (μg/L)	6.17 ± 4.12	56.8 ± 53.8	<0.001
Iron (mg/L)	29.4 ± 101	51.8 ± 52.1	<0.001
Manganese (mg/L)	0.418 ± 0.494	1.29 ± 1.51	<0.001
Nickel (μg/L)	38.5 ± 26.5	110 ± 79.8	<0.001
Calcium (mg/L)	67.6 ± 17.3	141 ± 89.3	<0.001
Magnesium (mg/L)	48.0 ± 23.4	91.8 ± 42.8	<0.001
Potassium (mg/L)	195 ± 94.4	242 ± 137	0.00194
Sodium (mg/L)	469 ± 226	736 ± 422	<0.001

^*a*^ Benjamini-Hochberg corrected *P* values are presented for Wilcoxon rank-sum comparisons between age classes in the 5 years prior to sampling. Only parameters that were significantly different at *P* < 0.05 are presented here. Remaining variables can be seen in Table S3.

The 5-year window was selected to represent the geochemical conditions that had most recently shaped the microbial communities examined. It remains possible that earlier conditions have exerted a long-term impact on community membership and functional capacity. Leachate concentrations of most metals (Cd, Cr, Co, Cu, Fe, Mn, Ni, and Zn) were significantly higher (*P* < 0.05) in Older cells at the time of filling relative to the time of sampling (Table S3). For example, concentrations of Cr in leachate decreased from 160 ± 410 to 6.17 ± 4.12 µg/L from filling to the five years prior to sampling, over 25 times lower. Similarly, Fe also decreased to over 25× less, from 849 ± 2,960 to 29.4 ± 101 mg/L (Table S3). Microbial communities in Older cells also had significantly higher abundance of functional genes and gene groups associated with resistance to some of these metals (Cr, Cd, Cu, Co, and Zn) and Fe oxidation and reduction, pointing to a possible link between historic metal concentrations and present-day microbe-metal interactions.

**Fig 4:**
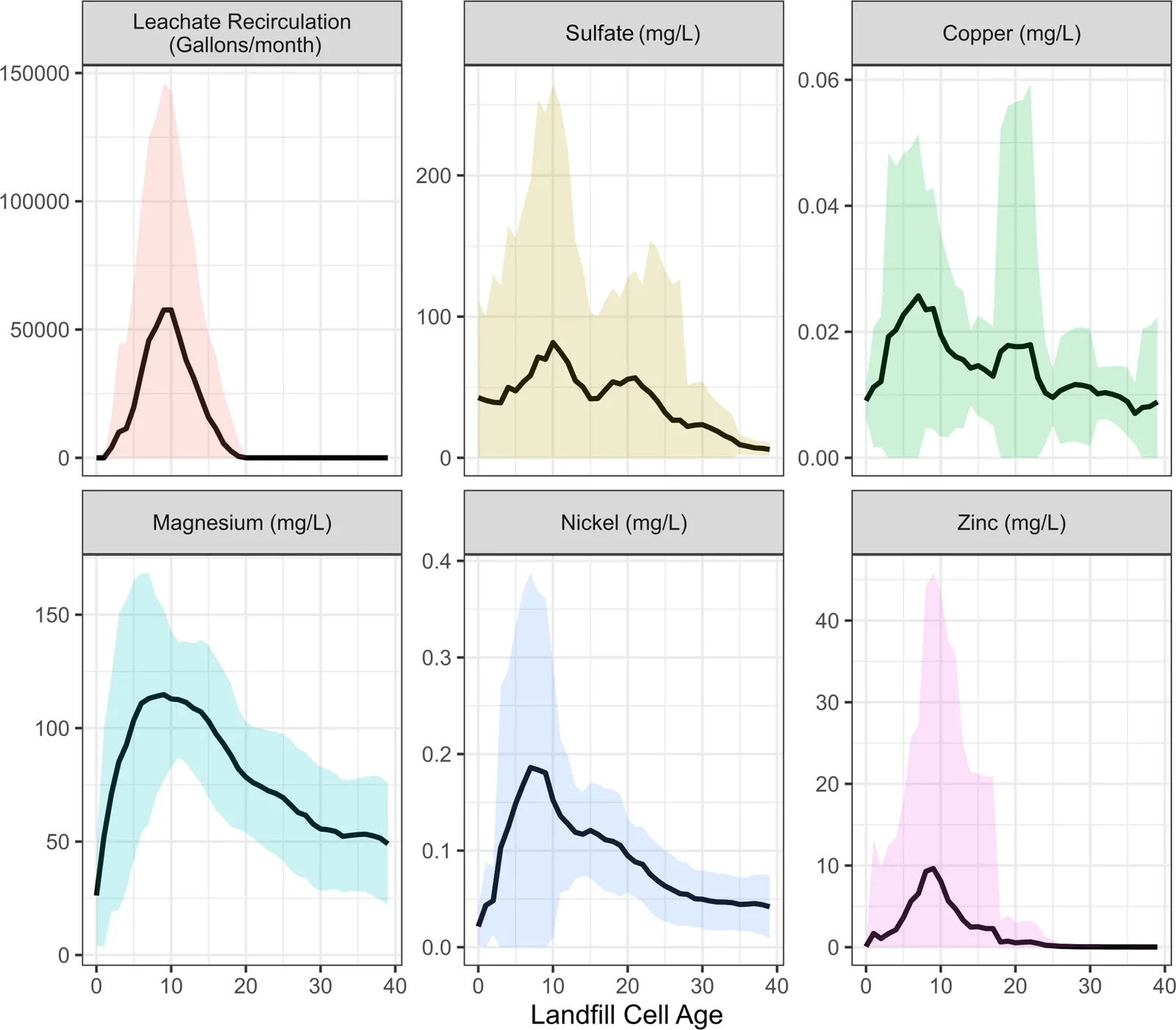
Line graphs depicting the 5-year rolling average (line) and standard deviation (shaded band around line) for landfill leachate parameters across 36 years of leachate chemistry data. All depicted parameters were within one k-means cluster (cluster 1).

To develop a picture of changes in overall landfill chemistry over decades, we aggregated leachate chemistry data into 5-year rolling averages. We computed *z*-scores for each parameter to assess differences in trends over time and clustered parameters into eight k-means clusters based on an elbow plot of within-cluster sum of squares (Fig. S4 and Table 2) to combine leachate chemistry variables into clusters with similar variance over time. We separated these clusters into those associated with key explanatory parameters (conductivity, redox, pH, OM breakdown) and remaining chemical groupings, highlighting changes over time by plotting averaged *z*-scores (Fig. 4; Fig. S5 through S9). This allowed us to identify key chemical parameters driving changes in landfill conditions. A table of all *z*-score-adjusted and averaged cluster data is available on the Open Science Framework (https://doi.org/10.17605/OSF.IO/MSDB8) (18).

**TABLE 2:** Cluster membership for 8 k-means clusters of *z*-score adjusted 5-year rolling averages of 36 years of landfill leachate data

Cluster no.	Key parameter	All parameters
1	Leachate recirculation	Leachate recirculation, sulfate, Cu, Mg, Ni, Zn
2	Organics and metals	BOD-5, COD, TOC, organic acids (acetic acid, propionic acid, isobutyric acid, butyric acid, isovaleric acid, and valeric acid), total phenolics, Ca, Cd, Cr, Fe
3	Redox	Redox (oxidation-reduction potential)
4	Conductivity	Conductivity, sulfide, ammonia-N, bicarbonate, As, K, Na
5	pH	pH, Mn
6	Nitrite-N	Nitrite-N
7	Nitrate-N	Nitrate-N, Se
8	Cobalt	Co

We found that a group of parameters (SO_4_^2−^, Cu, Mg, Ni, and Zn) clustered with the historic practice of leachate recirculation at the site, co-varying over time, with all parameters peaking in concentration at around 10 years since cells began filling (Cluster 1, Fig. 4). Leachate recirculation occurred off and on in the landfill from 1985 to 2009. The volume of leachate recirculated within each cell was strongly negatively correlated with precipitation inputs (*r*_s_ = 0.81, *P* < 0.001), suggesting that recirculation was performed to maintain reducing conditions in the landfilled waste during periods of low precipitation. To investigate the impact of leachate recirculation as a driver of leachate chemistry, we performed simple linear regressions on *z*-score-scaled 5-year rolling averages for all cell ages where there was leachate recirculation (Table S4). Parameters significantly influenced (*P* < 0.05) by the amount of recirculated leachate included carbon-associated parameters (BOD_5_ and COD), some metals (Cu, Cr, Fe, Mg, Ni, and Zn), oxidation-reduction potential, and two redox-associated nutrients (nitrite-N, sulfate). Given that leachate recirculation is performed to maintain reducing conditions that speed up waste decomposition, increases in BOD_5_/COD and decreases in oxidation-reduction potential are to be expected. The increases in metal concentrations may be linked to accelerated breakdown and release of labile OM, mobilizing more metals into solution, in addition to metals being recirculated back into the system.

The largest cluster contained parameters associated with the breakdown of carbon sources (BOD-5, COD, TOC, SCFAs, and total phenolics) and some metals (Ca, Cd, Cr, and Fe) (Cluster 2, Fig. S5). Calcium concentrations and carbon parameters all followed a similar trend, broadly peaking just prior to the 10-year mark then declining (Fig. S5). For Cd, Cr, and Fe, the rise and fall were more gradual, but still present. The peak for this cluster was earlier than the peak for the recirculation-associated group (including Cu, Mg, Ni, and Zn) (Table 2; Fig. 4 and Fig. S5). Contrastingly, oxidation-reduction potential (sole parameter in Cluster 3) showed a broad dip at 10 years and a slow rise afterward, reflecting potential oxygen infiltration into the aging landfill (Fig. S6).

Conductivity was clustered with sulfide, ammonia-N, bicarbonate, As, K, and Na (Table 2). Conductivity and concentrations of ammonia-N, bicarbonate, K, and Na all followed a similar trend with a broad peak between 10 and 20 years (Fig. S7). Sulfide and As were less similar, with a bump in concentration just before the 20-year mark (Fig. S7). pH only clustered with Mn, which both peaked at the beginning of the landfill life cycle before dropping off (Fig. S8).

The remaining clusters, all depicted in Fig. S9, were cobalt alone, nitrite-N alone, and a cluster containing nitrate-N and selenium. Trends in these were more sporadic over time, and total concentrations tended to be low (below 5 mg/L for nitrate-N and nitrite-N and below 1 mg/L for Co and Se).

Results for redox-associated parameters can be explained in the context of redox metabolism broadly. Manganese peaked early in the landfill life cycle, followed by Fe, both of which would likely be released into landfill leachate upon reduction from oxide forms. Then, following the large peak in Fe, there was a small increase in sulfide. This redox cascade is expected, though measured sulfide concentrations were quite low (<15 mg/L).

## DISCUSSION

**Fig 5:**
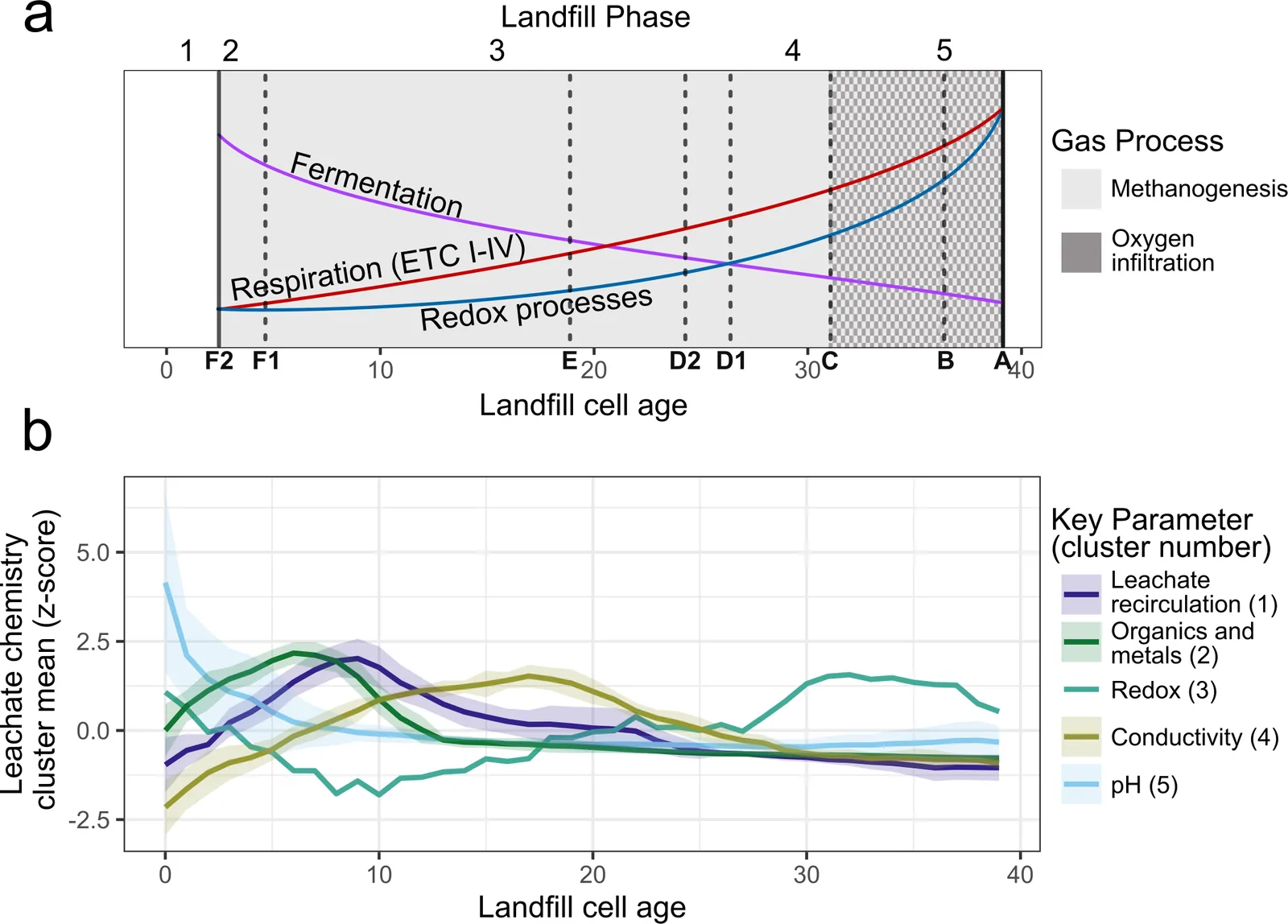
Conceptual diagram of landfill processes over time and landfill phases. (**a**) Trends in functional gene groups over landfill cells varying in age from 3 to 39 years across four landfill phases, overlaid on predicted methane/O_2_ dynamics. Solid lines represent the beginning and end of our observations, and the dashed lines represent the ages at which all other cells were sampled. Landfill phases are depicted along the top, but it is notable that cells F1 and F2 (the two newest cells) were transitioning from phases 2 to 3. Trend lines represent three functional gene groups: Fermentation (SCFA/alcohol degradation, carbohydrate-active enzymes), respiration (defined by abundance of complete [>70% of constituent genes present] electron transport chain complexes for ETC I–IV), and redox processes (comprising genes for redox transformations of Fe, N, S, and As). Gas dynamics are depicted as grayscale background, with methane dominating an anoxic environment from years 3 to 26, followed by oxygen infiltration in later years. (**b**) Line graph depicting trends in key geochemical variables across 36 years of leachate chemistry data. Lines depict the means of all z-score-adjusted parameters within a given k-means cluster, with standard deviation of all z-score-adjusted parameters within a cluster indicated by lighter bands. Clusters are named in the legend based on their key physical variable(s). Note that redox has no standard deviation because it was the sole member of its cluster (Cluster 3).

This study investigated differences in geochemistry and microbial community structure, composition, and functional genes in leachate from cells in a municipal solid waste landfill across 39 years of waste deposition. Few studies have explored the chemical and biological conditions in landfill cells in the later phases of decomposition under field conditions (phases 4 and 5) (3, 14). We compared “Older” (aged 31–39, phase 4/5) cells and “Newer” (aged 3–20, phase 2/3) cells, finding significant differences in microbial community structure and composition as well as microbial functional genes, and connected these differences to the underlying leachate chemistry. To anchor this discussion, we developed a conceptual model synthesizing results from microbial community functional predictions and landfill chemistry modeled over time (Fig. 5).

### Microbial contributions across the five-phase model of the landfill life cycle

This study fills important gaps in our understanding of the role microbial assemblages play in driving leachate chemical changes over the landfill life cycle, particularly in the later phases. In the early phases of the landfill life cycle, one of the key changes is an accumulation of labile OM (e.g., organic acids) as microbes break down complex organic material (3, 13). We observed a gradual increase in leachate organic acids (acetic, propionic, butyric, isobutyric, isovaleric, and valeric acid), biological oxygen demand, and chemical oxygen demand from 0 to 7 years for landfill filling, before a decrease of these parameters to near zero by year 12 (Fig. 5b—cluster 2). Similar trends in organic acids and BOD/COD have been found by other authors, who have observed major decreases within 10 years (13) or as little as 4 years (5). Within these first 7 years, pH declined substantially due to this accumulation of organic acids, produced from hydrolysis and fermentation of OM during the anaerobic acid phase (phase 2) of the landfill life cycle (3–5). It is expected that organic acids produced in phase 2 then feed the rapid methanogenesis that characterizes phase 3, explaining the overall decline in organic acids and biological and chemical oxygen demand (4).

Our microbial samples, collected in 2019, captured two cells transitioning from phases 2 to 3 (F1 and F2) and one in phase 3 (E), making up our group of “Newer” cells. These cells represent the early stages of landfill development, with communities structured around fermentation, reflected in the dominance of the families Dysgonomonadaceae and Cloacimonadaceae (Fig. 1b). MAGs from both families encoded a variety of genes associated with CAZyme families and with breakdown of SCFA and alcohol conversions. Members of the Dysgonomonadaceae have been found in a variety of environments, including landfills (27–29) and anaerobic digesters (30, 31), and are associated with the breakdown of a variety of organic compounds that are commonly present early in a landfill life cycle. In our data, the genomes of members of the Dysgonomonadaceae encoded genes for most of the investigated CAZymes as well as for conversions of butyrate, acetate, and pyruvate. Members of the Cloacimonadaceae have also been found in landfills (32) and anaerobic digesters (33, 34) and are associated with syntrophic propionate oxidation (34). Genomes from the Cloacimonadaceae contained fewer genes for CAZymes, targeting primarily chitin and starch degradation. The Cloacimonadaceae was also predicted to be active in acetogenic metabolisms, which likely support downstream methanogenesis (1, 32).

The abundance of genes associated with fermentative processes was significantly higher overall in Newer cells. This included CAZymes for the breakdown of many polysaccharides and genes associated with SCFA and alcohol conversion (Fig. 2). Further supporting the predominance of fermentative metabolism, Newer cells had a near absence of genes associated with complexes I–IV of the electron transport chain, which are necessary for respiration (Fig. 5).

During phase 3 (represented by Newer cells), rapid methanogenesis is the dominant process, utilizing breakdown products from the anaerobic acid phase (e.g., SCFAs). Methane was generated in all Newer cells, with large decreases in total gas flared in Intermediate and Older cells relative to Newer cells (summarized in detail in Grégoire et al. [1]). In keeping with this, Grégoire et al. (1) found a higher proportion of putative methanogens in Newer and Intermediate cells than in Older cells. Supporting this shift in microbial metabolism, we also observed large decreases in BOD, COD, TOC, and SCFAs, consistent with microbial consumption of labile carbon sources and a transition from fermentation-based processes (phase 2) to methanogenesis early in the landfill life cycle (phase 3) (Fig. 5).

During phase 4, total gas production tends to decrease, but methanogenesis proceeds (4, 7, 35). Reduced concentrations of SCFAs and other more labile carbon substrates by this phase lead microbes to rely on degradation of more complex OM, a slower process that reduces overall methane production. In our data, Intermediate-aged cells (D1 and D2) had more recently entered phase 4, while Older cell C was also classified to this phase. In all phase 4 cells (C and D), total gas production was substantially lower than in phases 2 and 3 (Newer cells) but still dominated by methanogenesis. We also observed a decrease in the abundance of genes or functional gene groups associated with fermentative functions and a corresponding increase in MAGs carrying genes that confer metabolic flexibility across a variety of redox processes.

Cell C exhibited higher abundance of functional genes and gene groups associated with redox cycling of Fe, N, and S than D1/D2, reflecting the shift toward metabolic flexibility by the end of phase 4, as methanogenesis continues to slow (Fig. 3 and 5). Interestingly, we also observed overall lower leachate concentrations of N, S, and Fe by phase 4, indicating a shift in microbial metabolism to lithoautotrophy as methanogenesis slowed and conditions became less reducing (Fig. 5b). These changes in functional potential were in large part due to shifts in microbial community composition between cells D1/D2 and cell C. Cell C, representing a later portion of phase 4, was broadly dominated by two MAGs from the genus *Aliarcobacter* (44.0% relative abundance). In particular, the genomes of these MAGs encoded genes related to denitrification (nitrate > nitrite > nitric oxide), N fixation, degradation of carbon sources (polyphenolics, chitin, and mixed-linkage glucans), oxidation of thiosulfate, and several genes related to acetate metabolism (acetate kinase, phosphate acetyltransferase, acetate--CoA ligase [ADP-forming] subunit alpha). The potential for metabolic flexibility, something documented across the Arcobacteraceae family (36, 37), may explain the predominance of this group in cell C. This metabolic flexibility may give members of this family a competitive advantage as biological activity declines throughout phase 4.

Phase 5 of the landfill life cycle, called the stabilization or maturation phase by some and the humic phase by others, is characterized by an increased potential for oxygen buildup and oxidative processes, as well as OM that is largely recalcitrant (e.g., humic and fulvic acids) (6–8). Because this phase may take decades to develop, few studies have investigated microbial communities and leachate geochemical conditions in detail. By phase 5, we observed an overall decline in carbon-associated parameters in leachate, including BOD, COD, TOC, and concentrations of SCFAs. This decrease in BOD, COD, and the BOD to COD ratio is often seen as indicative of the transition to “mature” landfill status (3, 5, 35). These declines also signify reduced overall biological activity, indicating reductions in rates of decomposition as landfill waste ages—a critical indicator of the stabilization phase. This reduction in decomposition may be linked to the observed increase in oxygen flared from Older cells at this site, indicating that a lower proportion of oxygen infiltrating the landfill is being consumed by microbes (1). This observed increase in oxygen, though still at low levels, has been proposed by several authors as an additional indicator of the stabilization phase (3, 4). We also observed an increase in oxidation-reduction potential across this window, indicating a shift toward more oxidizing conditions, driving changes in microbial metabolism and geochemical transformations in the later phases of the landfill life cycle.

Reflecting the decrease in biological oxygen demand, we observed fewer overall MAGs and lower diversity in Older cells (A, B, and C) relative to Newer cells. Microbial communities in Older cells were relatively enriched in chemolithoautotrophs (e.g*.,* Gallionellaceae, Sulfurimonadaceae), contrasting with the variety of diverse putative heterotrophs observed in Newer cells. This shift from chemoorganoheterotrophs to chemolithoautotrophs was also observed by Sekhohola-Dlamini et al. (14), who found that a 14-year-old MSW landfill with higher total carbon and N was dominated by copiotrophs, while a nearby 36-year-old landfill was dominated by lithotrophs (14). Taxonomic shifts were also reflected in differences in functional gene abundances between Older and Newer cells. As noted above, the relative abundance of MAGs with genes associated with the fermentation of carbon sources was significantly lower in Older cells. Possibly associated with increases in oxygen availability, significantly more MAGs in Older cells had genes associated with electron transport chain complexes I–IV, indicating the potential for aerobic respiration under changing redox conditions. Furthermore, Older cells had more metabolically flexible microbial communities, with significantly higher relative abundance of MAGs with genes for reduction and/or oxidation of Fe, N, and S. For example, members of the genera *Gallionella* and *Sideroxyarcus* (formerly *Sideroxydans*), which were enriched in Older cells, are widely known to oxidize ferrous iron (38–40). Similarly, members of two other enriched genera, *Sulfuricurvum* and *Sulfurimonas*, contain members known to be metabolically flexible, capable of utilizing a variety of reduced S compounds as electron donors and nitrate or oxygen as electron acceptors (41–43). Concentrations of nitrate, nitrite, and sulfide in leachate were all relatively low (generally <5 mg/L across all time points), indicating either low levels available in the landfilled waste, steady conversion/uptake rates by the microbial community, or both. In contrast, concentrations of sulfate were relatively higher (>10 mg/L) throughout the landfill life cycle, with no significant differences between Newer and Older cells at the time of sampling.

### Redox response across decades of landfill waste decomposition

Ammonia-N, sulfate, and Fe concentrations in leachate spiked at different points in the landfill life cycle. Each of these redox-associated metabolites was linked to different biogeochemical cycles in our data set. First, other studies have reported a spike in ammonia-N midway through the landfill life cycle as organics are depleted, mobilizing N via hydrolysis of proteins in OM (3, 5). We observed a similar trend, with ammonia-N broadly peaking with conductivity, bicarbonate, potassium, and sodium at around 20 years of landfill waste age (Fig. 5B—cluster 4). This peak in conductivity, bicarbonate, and base cations (K and Na) has been observed elsewhere. Conductivity has been observed to increase in young and intermediate-aged landfills as ions are released from the breakdown of organic material (3, 6, 44). Contributing to conductivity, bicarbonate tends to increase in leachate as degradation of organic material produces CO_2_, forming carbonic acid and ultimately, bicarbonate ions (44, 45).

A notable trend during the mobilization and then reduction in labile carbon substrates from 0 to 10 years was an increase in leachate concentrations of Fe, Ca, Cd, and Cr, with peaks occurring around 6–8 years. (Fig. 5b—Cluster 2). Although mobilization of these elements may be linked to their pH and redox-dependent natures (3, 8), it is important to note that pH generally remained above 6 in all samples at all time points, a phenomenon observed in other landfills’ anaerobic acid phase (15). It is therefore unlikely that mobilization of Fe, Ca, Cd, and Cr is entirely pH-dependent, as the measured pH was not acidic enough to release these metals into solution. Instead, it is possible that metals formed soluble complexes or colloids with OM, increasing leachate concentrations relative to the later parts of the landfill life cycle (3). Furthermore, reducing conditions in the anaerobic phases may have influenced metal mobility, reducing, for example, Fe(III) to soluble Fe(II) (15). Further investigation of the links between OM and metal mobility is needed; however, the enrichment of Fe-, N-, and S-cycling functional gene groups in Older cells suggests that microbially mediated redox transitions may contribute to metal fate over the landfill life cycle. Finally, concentrations of sulfate, Cu, Mg, Ni, and Zn were all linked to the historic practice of leachate recirculation at the site (Fig. 4 and 5b). Leachate recirculation is common in MSW landfills, where it is used to raise moisture content and hasten anaerobic decomposition (3, 45). At our site, leachate recirculation was practiced until the end of 2009, with variable volumes recirculated in each cell monthly. Because recirculation ceased in 2009, we had more data for leachate recirculation from Older and Intermediate cells (A, B, C, D1, and D2), with cell E only filling from 1999 onwards, and cell F not extant in 2009 (2014 start to filling). Linear regression analysis revealed that the volume of leachate recirculated was negatively associated with redox and nitrite-N and positively with BOD, COD, sulfate, Cu, Cr, Fe, Mg, Ni, and Zn over time. Outside of studies specifically investigating leachate recirculation in landfills (46, 47), few mention if the practice was performed at their sites or consider the broader impacts recirculation has on microbial guilds that control waste decomposition, making it difficult to put our results in context.

From our data, we suggest that more reducing conditions (decreases in redox potential) are an expected response to leachate recirculation, which is broadly associated with increased rates of fermentation of OM, possibly leading to higher BOD and COD (46, 47). Increased moisture content, brought about by either recirculation of leachate or precipitation, can also promote dissolution of metal-bearing minerals (e.g., Fe/Mn-oxides) and mobilize metals previously bound to solid phases—this is particularly true for metals like Cu, Ni, and Zn that readily dissolve in water or bind to organic ligands, thus keeping them in the leachate solution (15). Increased moisture content in landfill cells may also increase contact time, which can release a variety of ions from solid phases, including metals and sulfate. Additionally, it is unknown whether recirculated leachate at the site was exposed to the air prior to recirculation, which may have introduced additional oxygen to the solid landfill waste, promoting oxidation of sulfides. Although leachate recirculation was associated with lower ORP, oxidation and reduction of S compounds can occur simultaneously, as observed by Scholl et al. (48) in landfill-impacted groundwater (48). The occurrence of high sulfate concentrations in leachate despite reducing conditions may point to sulfide oxidation rates exceeding sulfate reduction rates within the solid material. Without samples from the landfill solid material, which we did not have access to, it is difficult to know for certain.

### Metal fate within a landfill

Given concerns about the impact of increasingly oxidizing conditions on metal mobility and metal leaching in landfills (4, 15), one of the goals of our study was to investigate changes in metal availability and putative metal cycling over the landfill life cycle. At the time of filling, metal concentrations were higher in A, B, and C (all filled before 1993) than in D1 and D2 (filled between 1993 and 1998), possibly due to changes in waste streams and waste management practices over time. We hypothesized that Older landfill cells would host microbial communities with a higher abundance of functional genes associated with utilization of inorganic electron acceptors and cycling of metals. We found that the abundance of metal resistance genes was significantly higher in Older cells relative to Newer. Many different genes for metal resistance (for As, Cd, Cu, Co, Cr, Fe, Ni, Se, and Te) were significantly more abundant in Older cells than Newer cells, despite lower metal concentrations. Genes for Fe oxidation and reduction were the only metal metabolism genes with significantly higher proportional abundances in Older cells. Interestingly, outside of Fe-cycling, the majority of metal resistance and metal cycling genes or gene groups were encoded by lower abundance MAGs that were not part of the most dominant families in Older cells (Gallionellaceae and Sulfurimonadaceae). Genes for arsenic resistance were some of the most ubiquitous in Older cells, present across a variety of taxa including many, but not all, Gallionellaceae and Sulfurimonadaceae MAGs, lineages known to harbor arsenic resistance genes in other environments (42, 49). Genes for arsenic resistance have been found across a variety of environments (23), including deep groundwater systems (49) and permafrost (50). While metal exposure may partially explain the high incidence of metal resistance genes in Older cells, which had the highest metal levels historically (51), some studies have seen little overlap between metal exposure and relevant metal-resistance genes (49, 52). It is possible that fluctuations in redox conditions within the solid material in Older landfill cells result in spatial differences in metal exposure that influence microbial community composition and functional gene profiles, but that is difficult to determine without sampling solid landfill waste. Our study provided a comprehensive look at a wide range of metals, and further study is needed to elucidate direct and indirect biogeochemical cycling pathways for landfill metals.

### Conclusions

In our study, we examined microbial communities, functional genes, and leachate chemistry across a landfill’s cells of varying ages (2–39 years). We also tracked changes in geochemical conditions across 36 years of landfill leachate chemistry data. We found that the early phases of the landfill life cycle were dominated by fermentative processes, with labile carbon declining over time. Conditions became more oxidizing over time, with evidence of oxygen buildup in Older cells. Microbial diversity declined with time, and there was a clear shift from fermentative processes to utilization of alternative electron donors and acceptors. Our results were largely consistent with studies that modeled potential biogeochemical cycles in landfills in the stabilization phase, having never recorded them (3, 4).

The transition away from fermentation-dominated metabolism to metabolic flexibility over the landfill life cycle was a key finding of our study. Our data suggest that the shift toward Fe-, N-, and S-cycling guilds in Older cells may mediate redox shifts that govern metal mobility, connecting OM cycling and metal fate in landfills. Landfill phase models have historically focused on fermentation and methanogenesis, but microbial respiratory and metabolic flexibility may also signal a shift in landfill biogeochemical processes as landfills approach stabilization.

Leachate metal concentrations were influenced by OM content and historic leachate recirculation practices. Leachate recirculation, which is rarely explored in landfill literature, was associated with increases in metal mobility and the potential for metal leaching out of the system, possibly linked to organo-metal complexes. Future research investigating organo-metal complexes and the ability of natural landfill decomposition to pull metals into solution would be an exciting new avenue to better understand metal fate in these environments, especially in conjunction with better modeling for how leachate recirculation-modified moisture regimes impact metal leaching and OM cycling.

Our model of the key processes and metabolisms over a landfill’s life cycle provides a new conceptual model of biogeochemical succession, based on empirical data, and synthesizing physical processes with microbial impacts. We note that this landfill’s cells were only at the beginning of the stabilization phase. The landfill should continue to be monitored for changes in geochemical conditions and microbial community succession over time to better understand the unexplored dynamics of this crucial final stage in a landfill life cycle.

## MATERIALS AND METHODS

### Site description

The sampled site is a municipal landfill located in the NEUS. The site is composed of six landfill cells (A, B, C, D, E, and F), two of which are divided into sub-cells (D into D1 and D2, and F into F1 and F2), each equipped with leachate collection systems. Previous work at this site classified the landfill cells using a five-phase conceptual model based on leachate chemistry and biogas data (1). Cell A, which received waste from 1980 to 1982, and Cell B, which received waste from 1982 to 1988, were both classified as in phase 5. Cell C, which received waste from 1988 to 1993, was classified into phase 4. The authors of this prior study identified evidence of oxygen intrusion into cells A and B, informing their classification into phase 5 of the landfill life cycle. The two subcells of cell D were filled at different times. D1 received waste from 1993 to 1998 and cell D2 from 1995 to 1998. Both were classified into phase 4, due primarily to low gas production relative to cells E and F. Cell E was filled from 1999 to 2014 and was classified into phase 3 (rapid methanogenesis) due to high gas output, mostly composed of methane. Cell F1 filled from 2014 up to the time of sampling (March 2019) and F2 from 2016 up to the time of sampling (March 2019). Both were identified as transitioning from phase 2 (anaerobic acid phase) to phase 3 due to variable gas production but still high proportions of methane (1). For this study, decreases in oxygen demand (BOD) and chemical oxygen demand (COD) at A, B, and to a lesser extent, C, relative to the other cells, prompted us to classify A, B, and C as “Older.” Cells E, F1, and F2 were categorized as “Newer,” while D1 and D2 were labeled “Intermediate.”

### Leachate sampling

In 2019, we collected leachate samples from each cell/sub-cell for shotgun metagenomic sequencing. One leachate monitoring well at each cell was purged of 2–3 well volumes, and 1 L of leachate was collected using a peristaltic pump. Leachate samples were stored on ice and transported to the lab before being filtered through a 0.22 μm Sterivex filter (Millipore Sigma, Burlington, MA) on the day of sampling. Filters were stored at −80°C until DNA extractions were performed. Detailed descriptions of DNA extraction, sequencing, filtering, assembly, binning, and taxonomic assignment can be found in Grégoire et al. (1), which exclusively explored the predicted methane-cycling populations across the landfill. The pipeline for microbial community analyses is described in brief below. Notably, no solid material was collected from the landfill, as collecting it would disrupt the landfill cap and disturb redox regimes and decomposition processes in the landfilled waste. Our study utilized leachate biogeochemical data to infer processes occurring in the solid waste.

### DNA sequencing and prior analyses

DNA was extracted from biomass on Sterivex filters using the PowerSoil DNA Isolation Kit (Qiagen) according to the manufacturer’s protocols but with the filter added to the initial bead tube in place of soil, and with elution into 50 µL of final buffer. DNA was quality-checked using a NanoDrop 1000 (Thermo Scientific, Waltham, MA) and quantified using Qubit fluorimetry (Thermo Scientific, Waltham, MA). Extracted DNA was sent to The Centre for Applied Genomics in Toronto, Canada, for shotgun metagenomic sequencing on an Illumina HiSeq machine with paired 2 × 150 bp reads. Acquired metagenomic reads were quality-trimmed with bbduk in the BBTools suite (https://sourceforge.net/projects/bbmap/) and Sickle v1.33 (53) prior to assembly using SPAdes3 v3.15.5 (54). Scaffolds were trimmed by length (≥2.5 kbp), and reads were mapped onto scaffolds using Bowtie2 v2.3.4.1 (55). Scaffolds were binned using three different binning algorithms (CONCOCT v0.4.0, MaxBin2 v2.2.6, MetaBAT2 v2.12.1), and bins were dereplicated using DAS Tool v 1.1.1 before quality-checking in CheckM v 1.0.13 (56–60). A total of 1,647 MAGs were retained with >70% completion and <10% contamination. Relative abundance of MAGs was calculated using the mean coverage for unique scaffold identifiers within each genome bin. To evaluate the functional potential of microbial communities, MAGs were annotated using DRAM v1.0 (21) to look at a wide array of functional genes using both sequence similarity and HMM-based detection.

### Data analyses

All previous work by Grégoire et al. is outlined in brief in “DNA sequencing and prior analyses,” above (1). For this study, taxonomy was assigned to MAGs using the Genome Taxonomy Database Toolkit (GTDB-tk) r266 (17). Phylogenomic analysis for all MAGs was performed using GToTree v1.6, using a suite of 25 HMMs for bacteria and archaea to identify single-copy genes and generate a tree (16). In total, 1,570 out of our 1,647 MAGs were placed on the tree. The resultant tree was visualized using the Interactive Tree of Life tool (61). To investigate similarity of MAGs across landfill cells, we utilized the dereplication tool dRep, which uses Mash and genome-wide ANI (gANI) to create clusters of MAGs based on pairwise comparisons, choosing a similarity cutoff of 99% (62).

The product file from DRAM was used to investigate the presence of genes associated with central metabolism, carbon cycling, nitrogen cycling, and sulfur cycling. DRAM combines genes into functional gene groups based on the presence of key genes. For example, for the function “acetate, pt 1” to be considered present, MAGs need to have genes for both phosphate acetyltransferase and acetate kinase. We utilized these DRAM categories in all cases except the SOX complex for oxidation of thiosulfate, which DRAM considers to be present if any *sox* gene (*A, B, C, D, X, Y,* or *Z*) is present. Researchers have demonstrated that the presence of *soxXA, soxB,* and *soxYZ* is necessary for partial oxidation of thiosulfate, with *soxCD* completing the reaction (63). Accordingly, we decided to apply a stricter threshold than DRAM, considering the combined presence of *soxA, soxB, soxX, soxY,* and *soxZ* as required to indicate the ability for partial or complete oxidation of thiosulfate.

DRAM also annotates metabolic pathways and multi-subunit complexes (e.g., ETC) based on the proportion of genes present. We considered a pathway or complex to be present when over 70% of genes were present. We utilized HMM-based pipelines for Fe and As metabolism and resistance, using FeGenie (22) for iron and Dunivin et al.’s arsenic resistance and metabolism pipeline (23). FeGenie uses a library of curated HMMs to group iron-related functional genes, while Dunivin et al.’s pipeline looks for individual marker genes using their HMM set. We also utilized BacMet version 2.0 for targeted annotation of metal-resistance genes by looking for homologs to experimentally confirmed genes from the BacMet database (24). Genes from the BacMet database were filtered to only those that were involved in metal resistance, excluding genes related to antibiotic resistance. Because BacMet and Dunivin et al.’s pipeline employ different methods, we retained hits from both, even if the genes overlapped (e.g., *arsB* from Dunivin vs. *arsB* from BacMet). Further information on genes contributing to functional groups can be found in the documentation for the tools listed above (Table S2).

All subsequent data analyses, including statistics and generation of figures, were performed in R version 4.3.1 (2023-06-16 ucrt) (64). We separated data into three categories for downstream analysis: Older cells aged 31–39 years (cells A, B, and C) and Newer cells aged 3–20 years (cells E, F1, and F2). D1 and D2 (aged 24–26) were considered Intermediate and were not included in statistical analyses. Base R was used for statistical tests. The packages “phyloseq” and “microeco” were used for generating Venn diagrams of MAG overlap among categories and for producing relative abundance bar graphs (65, 66). Relative abundance was calculated by summing the mean coverage for all MAGs at a given taxonomic level (e.g., at the Family level). Similarly, functional gene abundance was calculated using the relative coverage of MAGs that possessed a given functional gene, group, or pathway. Due to the variety of tools used for annotating functional genes, we collapsed data into binary presence-absence data for each gene, pathway, or gene group. Therefore, if three MAGs in a cell had coverages of 1%, 2%, and 3%, and each possessed at least one *arsA* gene, the “abundance” of the functional gene would be 6%. Wilcoxon rank-sum tests were used to determine differences in functional gene abundance and chemical parameters between Older and Newer cells. Finally, heatmaps of functional gene abundance were generated using the package “ggplot2” (67).

### Leachate chemistry

Thirty-six years of leachate chemistry records were provided by the site owners, with all analyses performed by Brickhouse Environmental consultants. While many parameters were provided by the site owners, we selected the following as relevant to microbial function and showing variance over time: pH, oxidation-reduction potential, electrical conductivity, bicarbonate, BOD, COD, TOC, sulfate, sulfide, ammonia-N, nitrate-N, nitrite-N, organic acids (acetic acid, propionic acid, isobutyric acid, butyric acid, isovaleric acid, and valeric acid), total phenolics, base cations (Ca, K, Mg, and Na), and metals (As, Cd, Co, Cr, Cu, Fe, Mn, Ni, and Se). As leachate chemistry has changed greatly over time for some cells, we reported chemistry both during the time of filling and in the 5 years prior to our sampling to capture shifts over time.

To investigate trends in geochemical parameters over time, we computed 5-year rolling averages across all cells by time since filling across all 36 years (year 0–36) of provided leachate data. Then, we computed *z*-scores for each parameter, transforming data to a mean of 0 and standard deviation of 1 so trends across parameters could be compared agnostic of units. Using an elbow plot of within-cluster sum of squares to determine the optimum number of clusters (Fig. S4), we clustered the z-score-adjusted data into eight k-means clusters (Table 2). Plots of 5-year rolling averages were developed using the “ggplot2” package in R (67). Finally, to investigate the impact of the historic practice of leachate recirculation on leachate geochemistry, we ran linear regressions on z-score scaled 5-year rolling averages for all cells across the time window for which there was active leachate recirculation (>5 gallons of leachate/month).

## Data Availability

All data involved in this research are publicly available. Sequencing data have been deposited to NCBI under the BioProject PRJNA900590, with raw reads available on the SRA database. Geochemical data, microbial functional annotations, and microbial taxonomy data are all available in the Open Science Framework (https://doi.org/10.17605/OSF.IO/MSDB8). All R code used for statistical analyses and to generate figures is also on the Open Science Framework.

## SUPPLEMENTAL MATERIAL

Supplemental material aem.00311-26-s0001.docx Tables S1 to S4; Fig. S1 to S9.

ASM does not own the copyrights to Supplemental Material that may be linked to, or accessed through, an article. The authors have granted ASM a non-exclusive, world-wide license to publish the Supplemental Material files. Please contact the corresponding author directly for reuse.
